# Rhodopsin Forms Nanodomains in Rod Outer Segment Disc Membranes of the Cold-Blooded *Xenopus laevis*

**DOI:** 10.1371/journal.pone.0141114

**Published:** 2015-10-22

**Authors:** Tatini Rakshit, Subhadip Senapati, Satyabrata Sinha, A. M. Whited, Paul S.-H. Park

**Affiliations:** Department of Ophthalmology and Visual Sciences, Case Western Reserve University, Cleveland, Ohio, Unites States of America; University of Western Australia, AUSTRALIA

## Abstract

Rhodopsin forms nanoscale domains (i.e., nanodomains) in rod outer segment disc membranes from mammalian species. It is unclear whether rhodopsin arranges in a similar manner in amphibian species, which are often used as a model system to investigate the function of rhodopsin and the structure of photoreceptor cells. Moreover, since samples are routinely prepared at low temperatures, it is unclear whether lipid phase separation effects in the membrane promote the observed nanodomain organization of rhodopsin from mammalian species. Rod outer segment disc membranes prepared from the cold-blooded frog *Xenopus laevis* were investigated by atomic force microscopy to visualize the organization of rhodopsin in the absence of lipid phase separation effects. Atomic force microscopy revealed that rhodopsin nanodomains form similarly as that observed previously in mammalian membranes. Formation of nanodomains in ROS disc membranes is independent of lipid phase separation and conserved among vertebrates.

## Introduction

Rhodopsin is the dim light receptor in rod photoreceptor cells of the retina that initiates vision upon photon capture. The receptor is embedded at high concentrations in rod outer segment (ROS) disc membranes. Rod photoreceptor cells are exquisitely sensitive and capable of detecting single photons [1]. While the high concentration of rhodopsin embedded in retinal membranes can help increase the probability of photon capture, the resulting crowded membrane environment may impede diffusion-mediated signaling events. A similar situation exists in photosynthetic membranes. High concentrations of pigments in the membrane maximize absorption of sunlight but a supramolecular protein organization within the membrane counteracts the effects of diffusion impedance within the membrane that would occur otherwise [2]. Rhodopsin likewise can attain a supramolecular organization, which can counteract the effects of diffusion impedance by providing a platform for efficient signaling [3, 4].

The visualization of membrane protein organization within native membranes under physiological conditions presents a challenge. Atomic force microscopy (AFM) meets this challenge by allowing high-resolution imaging of membrane proteins within the physiological context of a lipid bilayer and buffer conditions [5–7]. AFM of native ROS disc membranes has revealed that rhodopsin forms oligomers comprising rows of dimeric receptor. Oligomers of rhodopsin have been shown to arrange as a densely packed paracrystalline lattice or as nanoscale domains (i.e.,nanodomains) that are heterogeneous in size [8, 9]. Confirmation that rhodopsin is the protein species in nanodomains has come from single-molecule force spectroscopy and antibody labeling studies [10, 11].

The densely packed paracrystalline lattice arrangement of rhodopsin observed by AFM has been questioned for its physiological relevance because of a potential artifact caused by lipid phase separation in the membrane at the low temperatures in which samples are routinely prepared [12]. It must be noted that temperatures used to prepare samples for AFM are no different than those used to prepare ROS samples for biochemical studies, such as those used to obtain kinetic information on rhodopsin-mediated signaling events (e.g., [13, 14]). Aside from the first study using AFM to visualize the arrangement of rhodopsin in ROS disc membranes [9], densely packed paracrystalline lattices of rhodopsin have not been detected in subsequent studies. In subsequent studies, a densely packed crystalline lattice attributed to rhodopsin has been observed in what appears to be the rim region of a ROS disc [15, 16]. This assignment may be incorrect since rhodopsin is largely excluded from the rim region [17]. Since densely packed paracrystalline lattices of rhodopsin are rarely observed, this arrangement may not be physiologically relevant and may arise from artifacts related to storage at low temperatures [11].

In contrast to the densely packed paracrystalline lattices of rhodopsin, rhodopsin nanodomains are the arrangement most commonly observed by AFM and is conserved among mammalian species tested so far [8, 11, 15, 18, 19]. As stated earlier, the nanodomains comprise oligomeric rhodopsin forming rows of dimers [8]. Vitrified ROS examined by cryo-electron tomography have also revealed rhodopsin arranged as oligomeric rows of dimers scattered throughout the plane of the ROS disc membrane forming nanodomains [4]. Thus, the evidence points to nanodomains as the physiological organizing principle for rhodopsin in native membranes. Since nanodomains of rhodopsin have been observed in retinal membranes of mammalian species, it is unclear whether this type of organization is dependent on lipid phase separation effects caused by low temperatures. Nanodomains of rhodopsin are still observed in murine ROS disc membranes prepared and investigated by AFM at room temperature [15]. However, lipid phase separation still occurs to some extent in mammalian ROS disc membranes even at these temperatures [12].

To examine the possibility that the nanodomain organization of rhodopsin occurs as a result of lipid phase separation at low temperatures, ROS disc membranes from *Xenopus laevis* were investigated. Phase separation of lipids at low temperatures occurs only in ROS disc membranes from mammalian species and does not occur in membranes from cold-blooded frogs [20]. *X*. *laevis* is often used as an animal model system to study the function of rhodopsin and associated retinal diseases [21–27]. In addition to examining potential effects of lipid phase separation, the current study will also determine how similarly rhodopsin organizes in ROS disc membranes of *X*. *laevis* compared to that in mammalian membranes.

## Materials and Methods

### Ethics Statement

The Institutional Animal Care and Use Committee at Case Western University School of Medicine approved all animal studies reported in the current study. Mice were euthanized by CO_2_ and *X*. *laevis* were euthanized in a solution of tricaine. All efforts were made to minimize suffering.

### ROS Disc Membrane Preparation

All experimental procedures were conducted under dim red light conditions. Murine ROS disc membranes were prepared from the retinas of 12–15 C57Bl/6J mice (The Jackson Laboratory, Bar Harbor, ME) as described previously [19]. Frog ROS disc membranes were prepared from the retina of mature *X*. *laevis* (Nasco, Fort Atkinson, WI). Frogs were dark-adapted overnight before being sacrificed in a solution of 0.26% tricaine (Sigma-Aldrich, St. Louis, MO). All centrifugation steps for the preparation of frog ROS disc membranes were performed at 15°C and samples were stored at 15°C. Retinal tissue was obtained from 5–6 frogs and placed in 300 μl of 8% (vol/vol) OptiPrep (Sigma-Aldrich, St. Louis, MO) in frog Ringer’s buffer (3 mM HEPES, 111 mM NaCl, 2.5 mM KCl, 1.6 mM MgCl_2_, 1.0 mM CaCl_2_, and 10.0 mM D-Glucose, pH 7.8). The sample was vortexed at half-maximal speed for 15 s and then centrifuged at 100 × *g* for 30 s. The supernatant was removed and layered on a 20–40% (vol/vol) continuous gradient of OptiPrep in 12 ml of frog Ringer’s buffer. The pellet was resuspended in 300 μl of 8% OptiPrep in frog Ringer’s buffer and the steps described above were repeated five times. The gradient was centrifuged for 50 min at 26500 × *g*. Intact ROS migrated in a lower band about two thirds of the way from the top. The band containing intact ROS was collected and diluted threefold in frog Ringer’s buffer. The diluted ROS solution was centrifuged for 30 min at 26500 × *g*. The resulting pellet contained purified ROS. ROS were osmotically burst by resuspending the pellet in 1 ml of buffer A (2 mM Tris-HCl, pH 7.4) and incubating overnight at 15°C. The next day, the solution was centrifuged at 16100 × *g* for 5 min. The pellet was washed with buffer A three times by resuspension and centrifugation. The final pellet containing ROS disc membranes was resuspended in 50 μL of frog Ringer's buffer.

### AFM Imaging and Analysis

All AFM procedures were conducted under dim red light conditions. Murine samples were prepared for AFM as described previously [18, 19, 28]. *X*. *laevis* samples were prepared for AFM by adding 40 μL of ROS disc membranes (5–10 μg/mL) onto freshly cleaved mica and incubating for 10 min at room temperature. The mica was washed 3 times with 40 μL of frog Ringer's buffer to remove unadsorbed material. Adsorbed ROS disc membranes were imaged by AFM in imaging buffer (20 mM Tris-HCl, 150 mM KCl, 25 mM MgCl_2_, pH 7.8). ROS disc membranes preferentially adsorb on mica exposing the extracellular surface [10, 11]. The consequence of this preferential adsorption is that the covalently linked sugar groups at the amino terminal region of rhodopsin interfere with the AFM tip, thereby preventing the high resolution necessary to resolve individual rhodopsin molecules in most instances [8]. The resolution in these instances is sufficiently high, however, to distinguish nanodomains, which comprise oligomeric rhodopsin [8]. Nanodomains were imaged and analyzed in the current study.

Contact mode AFM at room temperature was conducted on a Multimode II atomic force microscope equipped with an E scanner (13 μm scan size) and silicon nitride cantilevers with a nominal spring constant of 0.06 N/m (DNP-S, Bruker Corporation, Santa Barbara, CA) as described previously [18, 19]. Tapping mode AFM at 37°C was performed on a 5500 atomic force microscope equipped with a 90 μm scanner (Keysight Technologies, Santa Rosa, CA) and silicon nitride cantilevers with a nominal spring constant of 0.24 N/m (DNP-S, Bruker Corporation, Santa Barbara, CA) as described previously [19]. Samples were maintained at 37°C using a Peltier temperature-controlled sample plate (0–40°C temperature range). AFM images were analyzed using the software SPIP (version 6.2, Image Metrology A/S, Hørsholm, Denmark) as described previously [19]. The height profile and histogram were generated using Prism 6 (GraphPad Software Incorporated, La Jolla, CA).

### SDS-PAGE

ROS disc membranes were resuspended in lithium dodecyl sulfate sample buffer containing 50 mM dithiothreitol (Expedeon Incorporated, San Diego, CA). Solubilized samples (0.75–1 μg of protein) along with molecular weight markers (Precision Plus Protein Kaleidoscope, Bio-Rad, Hercules, CA) were loaded onto a 4–12% Tris–Glycine precast gel (Life Technologies, Grand Island, NY) and electrophoresis was conducted. Gels were silver-stained to detect proteins.

## Results and Discussions

### Nanodomain formation is independent of adsorption on a solid substrate

**Fig 1:**
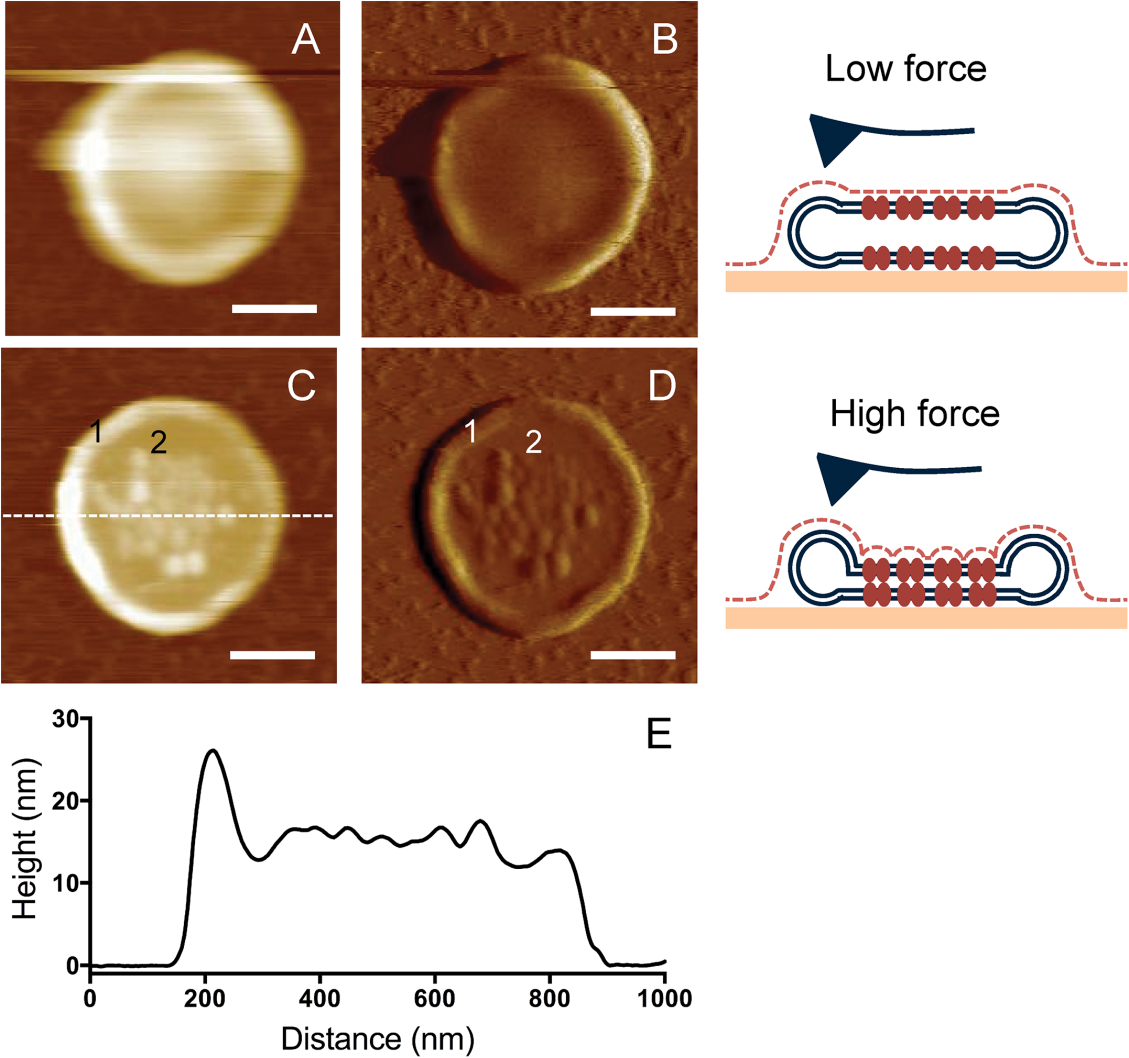
AFM image of an intact murine ROS disc. (A, B) Height (A) and deflection (B) images obtained by contact mode AFM generated using low force. (C, D) Height (C) and deflection (D) images obtained by contact mode AFM generated using higher force. The rim region (1) and nanodomains in the lamellar region (2) are discernible. Height images were scaled to a height range of 38 nm. Scale bar, 250 nm. Illustrations of a disc adsorbed on mica scanned by the AFM tip at low and high forces are shown next to AFM images. (E) A height profile is shown for the cross-section highlighted by a dotted line in panel C.

Samples of ROS disc membranes are first adsorbed on a mica substrate before investigation by AFM. Prior to examining a potential effect of lipid phase separation in ROS disc membranes, the possibility that nanodomains of rhodopsin are formed during the adsorption of samples on the mica substrate was examined. ROS discs are double bilayer membranes circumscribed by a rim region (Fig 1). As noted previously [18], a majority of discs adsorbed on mica and imaged by AFM are disrupted and only display a single bilayer membrane with a rim region. A minor fraction of discs, however, do adsorb on mica intact, which allowed for the imaging of the top membrane layer that is not in contact with the mica substrate.

An AFM image of an intact murine ROS disc collected using contact mode at low force showed no distinct morphology (Fig 1A and 1B). The top membrane is difficult to image due to flexibility of the membrane because it is not supported by a solid substrate. Applying higher force allowed for the resolution of the rim region and nanodomains in the lamellar region (Fig 1C and 1D), as is observed in ROS disc membranes with only a single membrane layer [18, 19]. The height of the nanodomains in the intact disc was about 16 nm (Fig 1E), which is double the height for nanodomains in a single bilayer ROS disc membrane [18]. The observation of rhodopsin nanodomains on the top membrane layer of an intact disc has been observed previously as well [16]. Thus, the formation of rhodopsin nanodomains is independent of adsorption on the mica substrate and are present in both single layered ROS disc membranes and intact discs.

### AFM of murine ROS disc membranes at physiological temperature

Characterization of rhodopsin nanodomains in ROS disc membranes has previously been conducted on samples prepared from mice at 4°C and imaged at room temperature [18, 19]. A nanodomain organization of rhodopsin has also been observed when samples were prepared and imaged at room temperature [15]. To determine whether nanodomain formation is related to possible lipid phase separation in the membrane at below body temperatures, we attempted to examine murine samples at 37°C. In initial studies, ROS disc membranes prepared from mice at 4°C were equilibrated to a temperature of 37°C prior to adsorption on mica and imaged by tapping mode AFM. No ROS disc membranes were observed by AFM, but instead, excess debris was observed indicating that ROS disc membranes are unstable at high temperatures.

**Fig 2:**
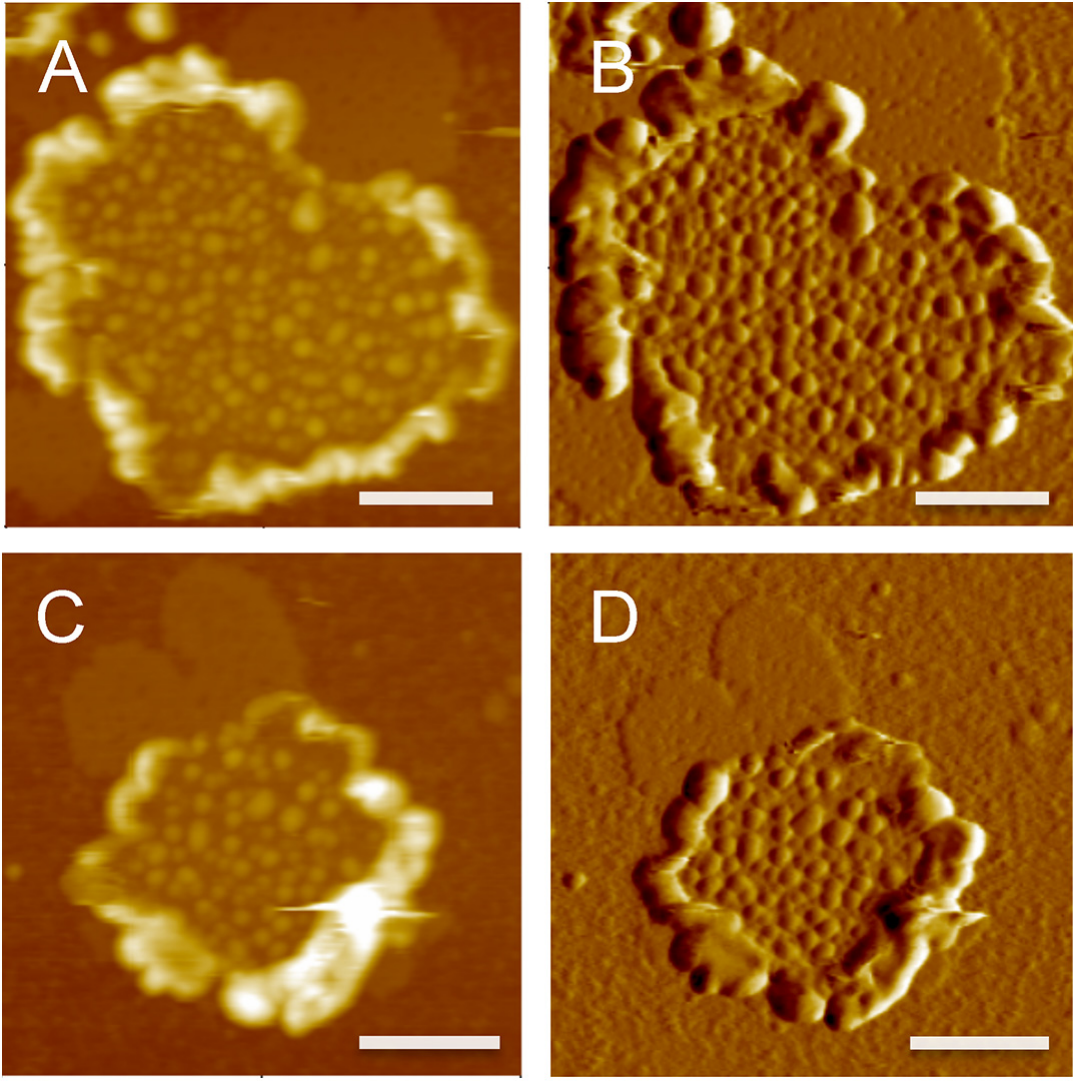
Murine ROS disc membranes imaged at 37°C. Representative images obtained by tapping mode AFM are shown. Murine ROS disc membranes were prepared at 4°C and imaged at 37°C. Height (left) and amplitude (right) images are shown. Height images were scaled to a height range of 25 nm. Scale bar, 500 nm.

Next, samples were adsorbed on mica at room temperature and imaged by tapping mode AFM at 37°C. Under these conditions, ROS disc membranes were observed (Fig 2), which indicates that the mica substrate stabilizes the membrane structure but does not induce the formation of nanodomains (e.g., Fig 1). ROS disc membranes displayed a single lamellar bilayer with a surrounding rim region. The lamellar region of the ROS disc membrane was organized into nanodomains, which is presumed to comprise oligomeric rhodopsin [8]. The overall morphology of ROS disc membranes observed at 37°C was similar as that observed previously with images obtained at room temperature [18, 19]. Thus, nanodomains of rhodopsin are still present when imaging is performed at 37°C. However, the instability of murine ROS disc membranes when incubated at 37°C prevented an explicit assessment of potential lipid phase separation effects on the formation of nanodomains.

### ROS disc membranes from *X*. *laevis*

**Fig 3:**
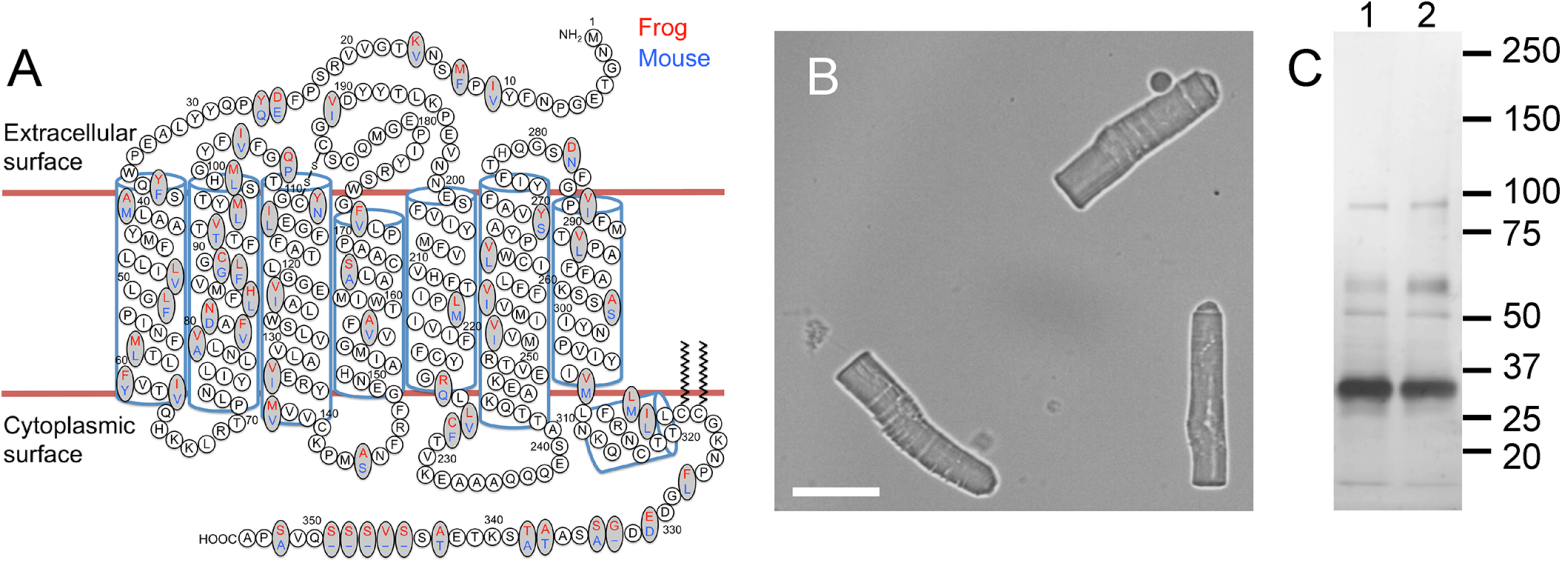
*X*. *laevis* ROS disc membrane preparation. (A) The secondary structure of rhodopsin is shown with amino acid residue differences in *X*. *laevis* (red) and murine (blue) rhodopsin highlighted. (B) Light microscopy image of purified ROS from the retina of *X*. *laevis*. Scale bar, 15 μm. (C) SDS-PAGE of *X*. *laevis* (lane 1) and murine (lane 2) ROS disc membrane preparations. The sizes of protein standards are indicated in kDa.

Since it was not possible to prepare and investigate murine ROS disc membranes at temperatures where lipid phase separation is absent, ROS disc membranes were prepared from the cold-blooded frog *X*. *laevis* and examined by AFM. Phase separation of lipids does not occur in ROS disc membranes of frogs at low temperatures [20]. Thus, *X*. *laevis* allowed for the preparation and investigation of samples by AFM at temperatures that preclude effects related to lipid phase separation in ROS disc membranes. A comparison of the amino acid sequences of rhodopsin from *X*. *laevis* and mice revealed that there are 61 amino acid residue differences (Fig 3A). Investigation of *X*. *laevis* ROS disc membranes by AFM additionally will reveal whether these differences in amino acid sequence impact the overall organization of rhodopsin in retinal membranes.

ROS were purified from the retinas of *X*. *laevis* and osmotically burst to release ROS discs. Purified ROS from *X*. *laevis* were considerably larger than those from mice (Fig 3B, cf. [18]). The ROS in frog retina is about 60 μm in length and 6 μm in diameter and that in murine retina is about 25 μm in length and 1 μm in diameter [16, 29–33]. Similar to murine ROS disc membranes, ROS disc membranes from *X*. *laevis* examined by SDS-PAGE resolved a single major band corresponding to rhodopsin (Fig 3C). Rhodopsin as the predominant protein species in frog ROS preparations has also been demonstrated previously by SDS-PAGE [34, 35]. Like murine ROS disc membrane preparations, *X*. *laevis* ROS disc membrane preparations contained predominantly a single protein species, rhodopsin, making it suitable for investigation by AFM.

**Fig 4:**
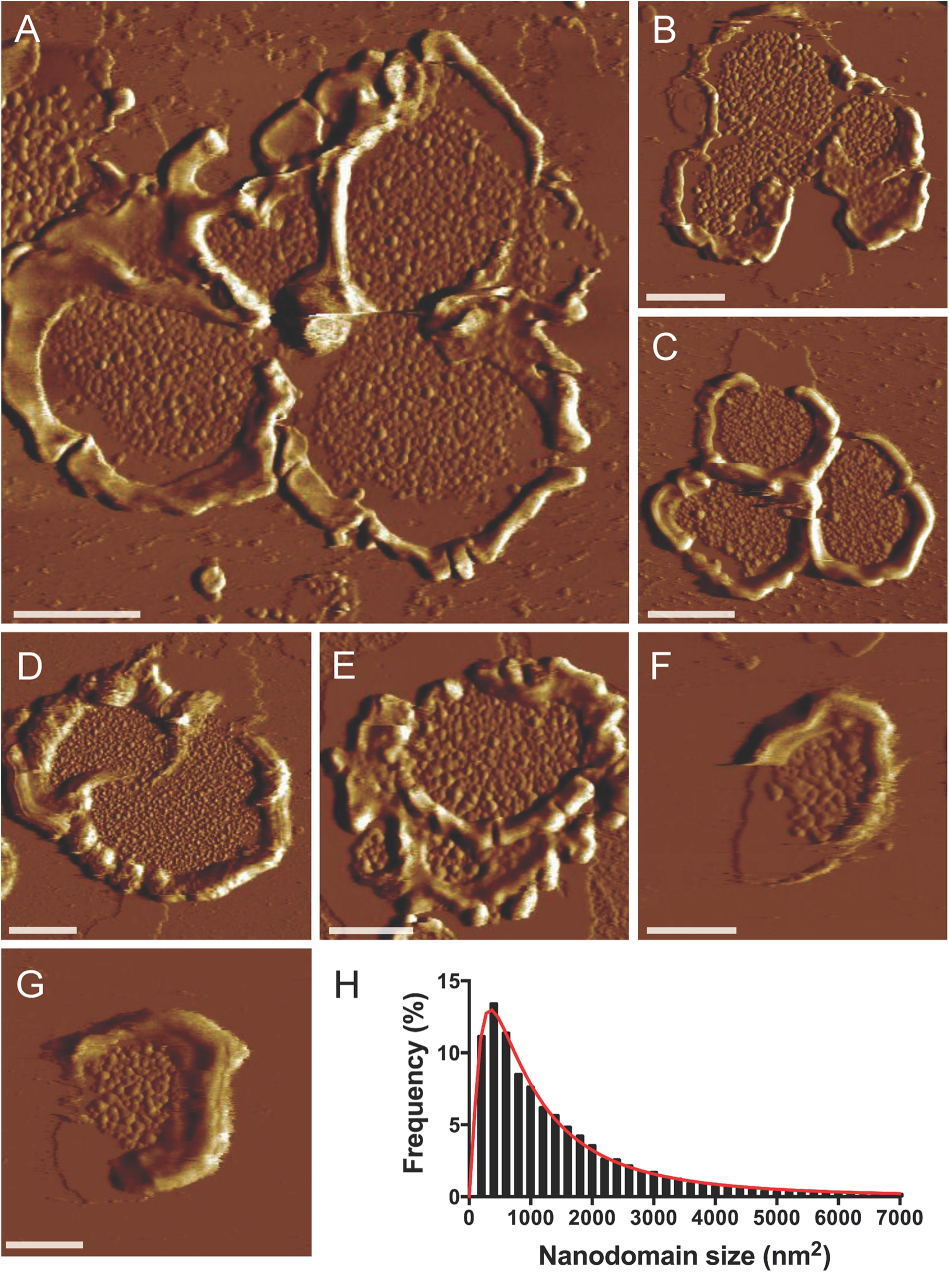
AFM images of *X*. *laevis* ROS disc membranes. (A-G) Representative deflection images of *X*. *laevis* ROS disc membranes obtained by contact mode AFM. ROS disc membranes exhibit a varying number of lobes, which are formed by deeply penetrating incisures. Scale bar, 500 nm. (H) Histogram of nanodomain sizes measured in 57 images of *X*. *laevis* ROS disc membranes. The data was fit by a Log Gaussian function (*n* = 14,390).

ROS disc membranes from *X*. *laevis* were adsorbed on mica and imaged by contact mode AFM at room temperature (Fig 4). Similar to ROS disc membranes of mice, samples from *X*. *laevis* exhibited a rim region and a lamellar region. The structure of frog ROS discs differs from that of murine ROS discs in size and number of incisures. Frog ROS discs are larger and contain several deeply penetrating incisures segmenting the disc into lobes [25, 32, 36, 37]. Murine ROS discs only have single incisures [4], which are fragile structures often disrupted in preparatory steps and infrequently observed by AFM [19]. Due to the fragility of incisures, ROS disc membranes from *X*. *laevis* adsorbed on mica were mostly fragments of an intact disc. A range of ROS disc membrane sizes were observed, with some exhibiting a single lobe (Fig 4F and 4G) and others exhibiting several lobes (Fig 4A–4E). Interestingly, ROS disc membranes with only a single lobe had a diameter of about 1.5 μm, which is similar in size to disc membranes from mice [18, 19]. Each lobe in ROS discs of *X*. *laevis* has been proposed to compartmentalize to some extent phototransduction signaling events [25]. Signaling events in each lobe may therefore be equivalent functionally to those occurring in a single murine ROS disc.

### Rhodopsin forms nanodomains in ROS disc membranes from *X*. *laevis*

Rhodopsin is present in the lamellar region of the ROS disc [17], where it is the predominant protein species (Fig 3C). Similar to murine ROS disc membranes in the lamellar region (Fig 2), the lamellar region of *X*. *laevis* ROS disc membranes exhibited nanodomains (Fig 4). The height of the nanodomains was about 8 nm, which is similar to the height of nanodomains in murine ROS disc membranes and corresponds to the height of a rhodopsin molecule [18, 38]. The sizes of the nanodomains were heterogeneous and histogram analysis revealed a Log Gaussian distribution (Fig 4H), which was similar to that observed for data from murine and human ROS disc membranes [18]. The size and density of nanodomains and the density of rhodopsin in a *X*. *laevis* ROS disc membrane were similar to that in murine ROS disc membranes [19]. The median nanodomain size in a single ROS disc membrane was 1,042 ± 295 nm^2^ (*n* = 57), which corresponds to 74 rhodopsin molecules assuming rhodopsin arranges as oligomers comprising rows of dimers as it does in murine ROS disc membranes [8]. Thus, the size of nanodomains reflects the oligomeric size of rhodopsin. The most frequently observed size of nanodomains was 350 nm^2^ (Fig 4H), which corresponds to 25 rhodopsin molecules. The density of nanodomains in ROS disc membranes was 203 ± 78 μm^-2^ (*n* = 57) and the density of rhodopsin was 21,008 ± 6,381 μm^-2^ (*n* = 57). The density of rhodopsin was computed based on estimates of the number of rhodopsin molecules contained within a nanodomain, as described earlier, and represents the density of rhodopsin if the receptor were homogeneously distributed within the plane of the membrane.

### Nanodomain organization of rhodopsin is conserved among vertebrates

The nanodomain organization of rhodopsin in ROS disc membranes has previously been observed in several mammalian species including humans, mice, and cows [8, 11, 15, 18, 19]. The amino acid sequences of rhodopsin among mammalian species are more similar to each other compared to the sequence of rhodopsin from *X*. *laevis*. The tertiary and quaternary structures of human, murine, and bovine rhodopsin are predicted to be quite similar [18, 39]. A comparison of murine and *X*. *laevis* rhodopsin reveals that there are 61 amino acid residue differences, with 14 non-conserved differences, 41 conserved differences, and 6 additional amino acid residues in *X*. *laevis* rhodopsin (Fig 3A). Despite these differences, rhodopsin in amphibian ROS disc membranes arranges similarly into nanodomains as it does in mammalian ROS disc membranes. The organization of rhodopsin into nanodomains in both mammalian and amphibian ROS disc membranes suggests that this organizing principle is conserved across all vertebrate species.

The observation of rhodopsin nanodomains in the ROS disc membranes of the cold-blooded frog *X*. *laevis* refutes the notion that nanodomains are formed as a result of lipid phase separation in the membrane. The phase separation that has been reported to occur in ROS disc membranes of mammalian species does not appear to impact the formation of rhodopsin nanodomains since the properties of nanodomains is similar in both mammalian and amphibian ROS disc membranes. Although lipid phase separation has been detected in mammalian ROS disc membranes, it must be noted that only a small fraction of lipids undergo phase transition and the disc membranes remain fluid even at temperatures approaching 0°C [20, 40]. If effects related to lipid phase separation exist, the overall organization of rhodopsin as nanodomains within ROS disc membranes appears to be largely unaffected by low temperatures routinely used in the preparation of ROS. Additionally, the formation of nanodomains is independent of disc disruption or adsorption on a mica substrate prior to conducting AFM. It has been proposed that incisures align dimeric rows of oligomeric rhodopsin in the membrane of photoreceptor cells [4]. If this type of alignment occurs, then the disruption of incisures could result in the misalignment of rhodopsin oligomers. Thus, while the formation of nanodomains itself appears to be independent of sample preparation and lipid phase separation, the position of nanodomains within ROS disc membranes observed by AFM may differ from that in live photoreceptor cells.

The observation of nanodomains in both ROS disc membranes by AFM and in disc membranes in intact ROS by cryo-electron tomography suggests that this is the native organization rhodopsin adopts in normal physiology [4, 8, 11, 15, 18, 19, 41]. The implications of this type of organization have begun to be considered computationally [3, 4]. This updated structural framework should be incorporated into our current view of phototransduction in photoreceptor cells and the determinants and role for this type of organization should be examined in more detail.
